# The interaction of AB-680, a CD73 inhibitor, with NBTI, a nucleoside transporter inhibitor, on the adenosinergic control of atrial contractility

**DOI:** 10.3389/fphar.2025.1683950

**Published:** 2025-10-21

**Authors:** Ignac Ovari, Agnes Boglarka Brezniczky, Attila Laczovics, Ervin Berényi, Tamas Erdei, Oluwatofunmi Ojo, Bence Hornok, Bela Juhasz, Zoltan Szilvassy, Judit Zsuga, Gabor Viczjan, Rudolf Gesztelyi

**Affiliations:** ^1^ Department of Pharmacology and Pharmacotherapy, Faculty of Medicine, University of Debrecen, Debrecen, Hungary; ^2^ Department of Radiology and Imaging Science, Faculty of Medicine, University of Debrecen, Debrecen, Hungary; ^3^ Department of Psychiatry, Faculty of Medicine, University of Debrecen, Debrecen, Hungary

**Keywords:** AB-680, CD73 inhibitor, nucleoside transport blocker, Guinea pig, atrium, A_1_ adenosine receptor, interstitial adenosine level

## Abstract

**Introduction:**

In this study, we investigated the influence of AB-680, a highly potent CD73 inhibitor, on the effect of NBTI, a nucleoside transport blocker, exerted on concentration-effect (E/c) curves generated with CPA, a relatively stable, selective A_1_ adenosine receptor full agonist, in isolated, paced guinea pig left atria.

**Methods:**

Transformations of the CPA E/c curves, constructed in the absence and presence of AB-680 and NBTI (in all combinations), were used to assess the changes in the interstitial adenosine concentration. These changes were quantified with the receptorial responsiveness method (RRM), a unique procedure providing the CPA concentration (as c_x_), which is equieffective with the increase in the interstitial adenosine concentration caused by NBTI. AB-680 and NBTI were dissolved in DMSO (recommended for *in vitro* use) as well as in a buffer (recommended for *in vivo* use), and the results were compared.

**Results and discussion:**

We found that AB-680, when added alone, did not affect the response to CPA. In turn, AB-680, when administered together with NBTI, was able to partially reverse the elevating effect of NBTI on the interstitial adenosine level. Nevertheless, the inhibitory action of AB-680 on the effect of NBTI appeared to be smaller than that of PSB-12379, another CD73 inhibitor investigated earlier in the same experimental model. We also found that DMSO interfered with our measurements to a lesser extent than the buffer recommended for *in vivo* studies. In addition, AB-680, when co-administered with NBTI (both dissolved in DMSO), reduced c_x_ (i.e. probably also the surplus interstitial adenosine) by at least half.

## 1 Introduction

Adenosine, in addition to its central role as a metabolite (precursor and degradation product of ATP), is an important humoral regulator in almost all tissues of higher organisms (Fredholm et al., 2001; 2011; Burnstock et al., 2010; IJzerman et al., 2022). The regulatory effects of adenosine are predominantly mediated by cell-surface adenosine receptors (A_1_, A_2A_, A_2B_, and A_3_); thus, it is the interstitial adenosine level that plays a key role in the adenosinergic control. In the myocardium, A_1_ is the main adenosine receptor type (Fredholm et al., 2001; Szentmiklósi et al., 2011; Headrick et al., 2013; Qian et al., 2025).

In the heart, the complex regulatory actions of adenosine occur predominantly during cellular energy (ATP) deficiency, a pathological condition characterized by an increase in the interstitial adenosine concentration (Szentmiklósi et al., 2011; Headrick et al., 2013; Qian et al., 2025). In this situation, the main source of interstitial adenosine is the intracellularly formed adenosine that is released from the cells *via* equilibrative nucleoside transporters, including ENT1, which is NBTI-sensitive (Schreieck and Richardt, 1999; Deussen et al., 1999; 2006; Deussen, 2000a; 2000b; Szentmiklósi et al., 2011; Burnstock and Pelleg, 2015).

In turn, under physiological circumstances, interstitial adenosine is primarily formed from ATP, which is released from the cells and then dephosphorylated to adenosine by ectonucleotidases, mainly by CD39 (ecto-apyrase) and consecutively by CD73 (ecto-5′-nucleotidase) (Hesse et al., 2017; Zimmermann, 2000; Qian et al., 2025; for a brief synopsis of ectonucleotidases, see Table 1 in Viczjan et al., 2021). In this condition, contrary to the energy-depleted state, the equilibrative nucleoside carriers transport adenosine into the cells because the adenosine formation exceeds the adenosine elimination in the interstitium (Deussen et al., 1999; Deussen, 2000a; Deussen, 2000b).

**TABLE 1:** Best-fit values (logc_x_) with their 95% confidence intervals (95% CI) and antilog values (c_x_) obtained with the receptorial responsiveness method (RRM). The c_x_ values, converted to nM (nmol/L), are the estimates for the interstitial adenosine accumulation in response to NBTI (as the equieffective CPA concentrations). RRM was implemented in two alternative ways: individual fitting (panels A and C) and two-model global fitting (panels B and D). The CPA E/c curves (in the three experimental groups named control, NBTI, and AB-680 + NBTI) were constructed in the presence of DMSO (panels A and B) or a buffer recommended for *in vivo* use (panels C and D) as solvents.

A	Individual fitting	
DMSO	NBTI	AB-680 + NBTI
logc_x_	−7.28	−7.61
95% CI	−7.39–−7.18	−7.88–−7.43
c_x_ (nM)	52.65	24.33
*R* ^2^	0.8994	0.9171
Adj. *R* ^2^	0.8994	0.9171

B	Two-model global fitting		
DMSO	Control	NBTI	AB-680 + NBTI
logc_x_	na	−7.56	−7.96
95% CI		−7.89–−7.32	−8.64–−7.56
c_x_ (nM)		27.39	10.93
*R* ^2^	0.9842	0.9092	0.9281
Gl. *R* ^2^	0.9384		
Adj. *R* ^2^	0.937		

C	Individual fitting	
Buffer	NBTI	AB-680+NBTI
logc_x_	−8.89	−9.72
95% CI	−9.61–−8.35	−10.55–−9.14
c_x_ (nM)	1.28	0.19
*R* ^2^	0.8062	0.9182
Adj. *R* ^2^	0.8062	0.9182

D	Two-model global fitting		
Buffer	Control	NBTI	AB-680+NBTI
logc_x_	na	−9.30	−10.12
95% CI		−10.3–−8.58	−12.92–−9.11
c_x_ (nM)		0.5	0.08
*R* ^2^	0.7435	0.8277	0.9303
Gl. *R* ^2^	0.8277		
Adj. *R* ^2^	0.822		

na, not applicable; CPA, *N* ^*6*^-cyclopentyladenosine; NBTI, S-(2-hydroxy-5-nitrobenzyl)-6-thioinosine; DMSO, dimethyl-sulfoxide; *R* ^2^, the coefficient of determination; Gl. *R* ^2^, the global coefficient of determination; Adj. *R* ^2^, the adjusted coefficient of determination.

Consistent with the above-mentioned well-established observations, we previously found that the inhibition of ENT1 by NBTI (S-(2-hydroxy-5-nitrobenzyl)-6-thioinosine) markedly elevated the interstitial adenosine level in the metabolically intact, isolated guinea pig left atrium (Karsai et al., 2006; 2007; Viczjan et al., 2021). On the conventionally constructed and plotted concentration–effect (E/c) curve of CPA (N^6^-cyclopentyladenosine), which is a relatively stable, selective A_1_ adenosine receptor full agonist (Fredholm et al., 2001; Pavan and IJzerman, 1998), this effect of NBTI manifested in a considerable decrease in the maximal effect (E_max_) and potency (1/EC_50_). This phenomenon was explained by the fact that the surplus interstitial adenosine accumulated by NBTI consumed a part of the response capacity of the A_1_ adenosine receptors during the incubation period with NBTI (i.e., before the construction of the E/c curve with the given A_1_ adenosine receptor agonist) (Gesztelyi et al., 2003; Karsai et al., 2006; Karsai et al., 2007).

We also found that PSB-12379 (N^6^-benzyl-α,β-methyleneadenosine-5′-diphosphate), a potent CD73 inhibitor (Bhattarai et al., 2015; Schmies et al., 2020), significantly inhibited the aforementioned effect of NBTI on the CPA E/c curve; i.e., it increased the response to CPA in the presence of NBTI (compared to the state when only NBTI was present). This phenomenon was attributed to the fact that PSB-12379 decreased the interstitial adenosine formation and, thereby, the ability of NBTI to increase the interstitial level of endogenous adenosine (in an energetically physiological condition) (Viczjan et al., 2021).

AB-680 is an extremely potent but slow-onset competitive inhibitor of CD73 (Bowman et al., 2019). The significance of AB-680 stems from its promise as an antitumor agent that appears to restore the antineoplastic activity of the immune system, which has been impaired by the high adenosine level characteristic of the interior of solid tumors (Piovesan et al., 2022; Strickland et al., 2024; Gao et al., 2025). However, to the best of our knowledge, no investigation has yet dealt with the effect of AB-680 on the heart or cardiac-derived tissues. Given the pivotal impact of the interstitial adenosine level on the heart, it is important to examine any agent that may also affect it in this regard.

In the present study, we aimed to investigate the action of AB-680 on the effect of NBTI exerted on the A_1_ adenosine receptor-mediated response, which is detectable with conventionally constructed and plotted CPA E/c curves, in the isolated, paced guinea pig left atrium. Transformations of the CPA E/c curves generated in the absence and presence of AB-680 and NBTI (in all combinations) were used to assess the changes in the interstitial adenosine concentration. These changes were quantified with the receptorial responsiveness method (RRM). RRM is a unique procedure providing the concentration of the agonist used for the E/c curve, which is equieffective with the surplus interstitial adenosine concentration compared to a predefined baseline (Gesztelyi et al., 2004; Grenczer et al., 2010a; Grenczer et al., 2010b). RRM was implemented according to the recommendations of our recent study (Ovari et al., 2024), performing both individual fitting and two-model global fitting.

The evaluation of the influence of AB-680 on atrial contractility, both alone and in combination with a nucleoside transport inhibitor, can provide insights into the quantitative relationships of the adenosinergic mechanisms in the atrial myocardium. In addition, these results might inform us about the nature and extent of some potential side effects of this CD73-inhibitor drug candidate in the heart.

## 2 Materials and methods

### 2.1 Materials

The following chemicals were used: adenosine, N^6^-cyclopentyladenosine (CPA), S-(2-hydroxy-5-nitrobenzyl)-6-thioinosine (NBTI), and dimethyl-sulfoxide (DMSO), which were purchased from Sigma-Aldrich (St. Louis, MO, United States); furthermore, quemliclustat (AB-680), polyethylene glycol with an average molecular weight of 300 (PEG 300), and polysorbate 80 (Tween 80) were obtained from MedChemExpress (Monmouth Junction, NJ, United States).

Adenosine was always dissolved in 36 °C modified Krebs–Henseleit solution (Krebs solution) containing (in mmol/L): NaCl (118), KCl (4.7), CaCl_2_ (2.5), NaH_2_PO_4_ (1), MgCl_2_ (1.2), NaHCO_3_ (24.9), glucose (11.5), and ascorbic acid (0.1) dissolved in redistilled water. CPA was dissolved in an ethanol: water solution (1:4 v/v, respectively). Both stock solutions were adjusted to a concentration of 10 mmol/L, and then they were further diluted with Krebs solution (when appropriate).

In the first series of experiments, AB-680 and NBTI were dissolved in DMSO, as recommended by the manufacturer of AB-680 for *in vitro* use (MCE, 2025).

In turn, in the second series of experiments, AB-680 and NBTI were dissolved in a buffer composed of DMSO, PEG 300, Tween 80, and normal saline (0.9% w/v of NaCl) in a ratio of 10:40:5:45 (v/v/v/v, respectively), as recommended by the manufacturer of AB-680 for *in vivo* use (MCE, 2025).

In the organ baths, the concentration of DMSO, PEG 300, and Tween 80 did not exceed 0.1%, 0.04%, and 0.005%, respectively (v/v), at any time.

### 2.2 Animals and groups

The animal use protocols were approved by the Committee of Animal Research, University of Debrecen, Hungary (11/2021/DEMÁB). Naïve, male Hartley guinea pigs with approximately 500 g and 400 g body weight (used for the first and second series of experiments, respectively) were obtained from Animalab Hungary Ltd. (Vác, Hungary), the distributor of Charles River Laboratories International Inc. (Wilmington, MA, United States). After a 2-week accommodation period, the animals were guillotined, and then the left atria were quickly removed. The isolated left atria were mounted at 10 mN resting tension in 10-mL vertical organ chambers (TSZ-04; Experimetria Ltd., Budapest, Hungary), which were filled with Krebs solution and gassed with 95% O_2_ and 5% CO_2_ (36 °C; pH = 7.4). The atria were paced by platinum electrodes (3 Hz, 1 m, twice the threshold voltage) using a programmable stimulator (ST-02; Experimetria Ltd., Budapest, Hungary) and a power amplifier (PST-02; Experimetria Ltd., Budapest, Hungary). The atrial contractile force was characterized by the amplitude of the isometric twitches, which were measured by a transducer (SD-01; Experimetria Ltd., Hungary) connected to a WS-DA-02 workstation (MDE GmbH, Heidelberg, Germany) using SPEL Advanced ISOSYS software (SOFT-02; MDE GmbH, Heidelberg, Germany). From the contractile force values, effect values were computed as a percentage decrease in the initial contractile force of the atria.

In the first series of experiments (using DMSO as the solvent), the atria were randomized into four groups: control (n = 6), AB-680 (n = 4), NBTI (n = 12), and AB-680 + NBTI (n = 7).

In the second series of experiments (using the complex buffer as the solvent), the atria were randomly divided into three groups: control (n = 6), NBTI (n = 7), and AB-680 + NBTI (n = 5).

### 2.3 Protocols

All atria were first allowed to equilibrate in Krebs solution for 25 min; subsequently, they were exposed to 100 μM adenosine for 2 min (as a priming), followed by a washout with Krebs solution for 20 min. Afterward, a cumulative E/c curve was constructed with adenosine on all atria, followed by another 20-min washout period (using Krebs solution). From this point, the experiments could continue in two ways.

In the first series of experiments, the control group received 10 μL DMSO for 30 min. The AB-680 group was subjected to 3 μM AB-680 (administered in 10 μL DMSO) for 30 min. The NBTI group received 10 μL DMSO for 15 min, and then (after a washout) it received 10 μM NBTI (added in 10 μL DMSO) for an additional 15 min. The AB-680 + NBTI group received 3 μM AB-680 (added in 10 μL DMSO) for 15 min, and then (after a washout) it received 3 μM AB-680 and 10 μM NBTI (co-administered in 10 μL DMSO) for an additional 15 min. Finally, a cumulative E/c curve was constructed with CPA for all the groups.

In the second series of experiments, the control group received 20 μL buffer for 30 min. The NBTI group received 20 μL buffer for 15 min, and then (after a washout) it received 10 μM NBTI (administered in 20 μL buffer) for an additional 15 min. The AB-680 + NBTI group received 3 μM AB-680 (added in 20 μL buffer) for 15 min, and then (after a washout) it received 3 μM AB-680 and 10 μM NBTI (co-administered in 20 μL buffer) for an additional 15 min. Finally, a cumulative CPA E/c curve was generated for all the groups.

### 2.4 Characterization of the E/c curves

The effect values were plotted against the corresponding agonist concentrations that were administered. These E/c curves were fitted to the Hill equation to illustrate them and obtain the values of the three empirical Hill parameters to describe the control E/c curves. The classical form of the Hill equation is as follows (Hill, 1910; Gesztelyi et al., 2012):

$$E=E_{\max}\cdot \frac{c^{n}}{c^{n}+E{C_{50}}^{n}},$$ (1)

where E is the effect, c is the concentration of the agonist in the bathing medium administered for the E/c curve [due to the short half-life of adenosine in the living tissues (Pavan and IJzerman, 1998; Fredholm et al., 2001), its concentrations in the organ bath and at the binding site of the adenosine receptors can significantly differ], E_max_ is the maximal effect (reachable by the agonist used), EC_50_ is the agonist concentration producing a half-maximal effect (reciprocal potency), and n is the Hill coefficient (slope factor).

For the regression with the Hill equation, the software’s built-in equation “log(agonist) vs. response-variable slope (four parameters)” was applied, with the “bottom” parameter constrained to 0 (GraphPad, 2025).

### 2.5 Quantification of the distortion of the CPA E/c curves caused by NBTI

The modification of the CPA E/c curves elicited by NBTI (alone or in the simultaneous presence of AB-680) was quantified using RRM. The core of RRM is described by the following relationship (Gesztelyi et al., 2004):

$$E^{\prime }=100-\frac{100\cdot \left(100-E_{\max}\cdot \frac{{\left(c_{x}+c\right)}^{n}}{{\left(c_{x}+c\right)}^{n}+E{C_{50}}^{n}}\right)}{100-E_{\max}\cdot \frac{{c_{x}}^{n}}{{c_{x}}^{n}+E{C_{50}}^{n}}},$$ (2)

where (adapted to the conditions of the present study) E’ is the distorted effect (an effect that was determined when NBTI was present), c is the CPA concentration in the bathing medium (administered for the E/c curve and, due to the relative stability of CPA, assumed to be equal to the CPA concentration at the binding site of the A_1_ adenosine receptors), E_max_, EC_50_, and n are the empirical Hill parameters defining the NBTI-naïve condition (upon individual fitting, these parameters were fixed at constant values obtained by fitting the Hill equation to the averaged CPA E/c curve of the appropriate control group, whereas upon two-model global fitting, these parameters were variable), and c_x_ is the parameter indicating the CPA concentration that is equieffective with the surplus interstitial adenosine concentration elicited by NBTI (thought to be the sole cause of the E/c curve modification compared to the control condition).

RRM was performed by implementing both individual fitting and two-model global regression (using equations equivalent with Equations 1, 2), as described by Ovari et al. (2024).

### 2.6 Data analysis

Curve fitting and statistical analysis were performed using GraphPad Prism version 10.5.0 (GraphPad Software Inc., La Jolla, CA, United States), while other calculations were carried out using Microsoft Excel 2024 (Microsoft Co., Redmond, WA, United States).

The distribution of data and homogeneity of variances were tested using the Shapiro–Wilk test and the Brown–Forsythe test, respectively. To compare groups with homogeneous variances and data following a Gaussian distribution, ordinary one-way ANOVA with Tukey’s post-test was carried out. Groups without homogeneous variances but with Gaussian data were compared using Brown–Forsythe ANOVA, followed by Dunnett’s post-test. To compare groups without homogeneous variances and Gaussian distribution, the Kruskal–Wallis test with Dunn’s post-test was carried out. Differences in means or medians were considered significant at *p* < 0.05.

The precision of the regression was characterized by the width of the 95% confidence interval (CI) of logc_x_ (the best-fit value that is most important for us). The precision of the curve fitting was indicated by the distance of the 95% confidence bands from the corresponding best-fit curve.

To check how well the experimental data define the model, the option “Identify “ambiguous” fits” was chosen (Graphpad, 2025) because, in general, more estimates could be obtained this way.

The goodness-of-fit was quantified by the coefficient of determination (*R* ^2^) and its adjusted value.

The default option for computing 95% CIs was almost always “asymmetrical.” When not (i.e., during the definition of the models to fit), the “asymmetrical” option was chosen. For every setting not addressed above, the default option was used (GraphPad, 2025).

## 3 Results

### 3.1 Initial contractile forces

**FIGURE 1:**
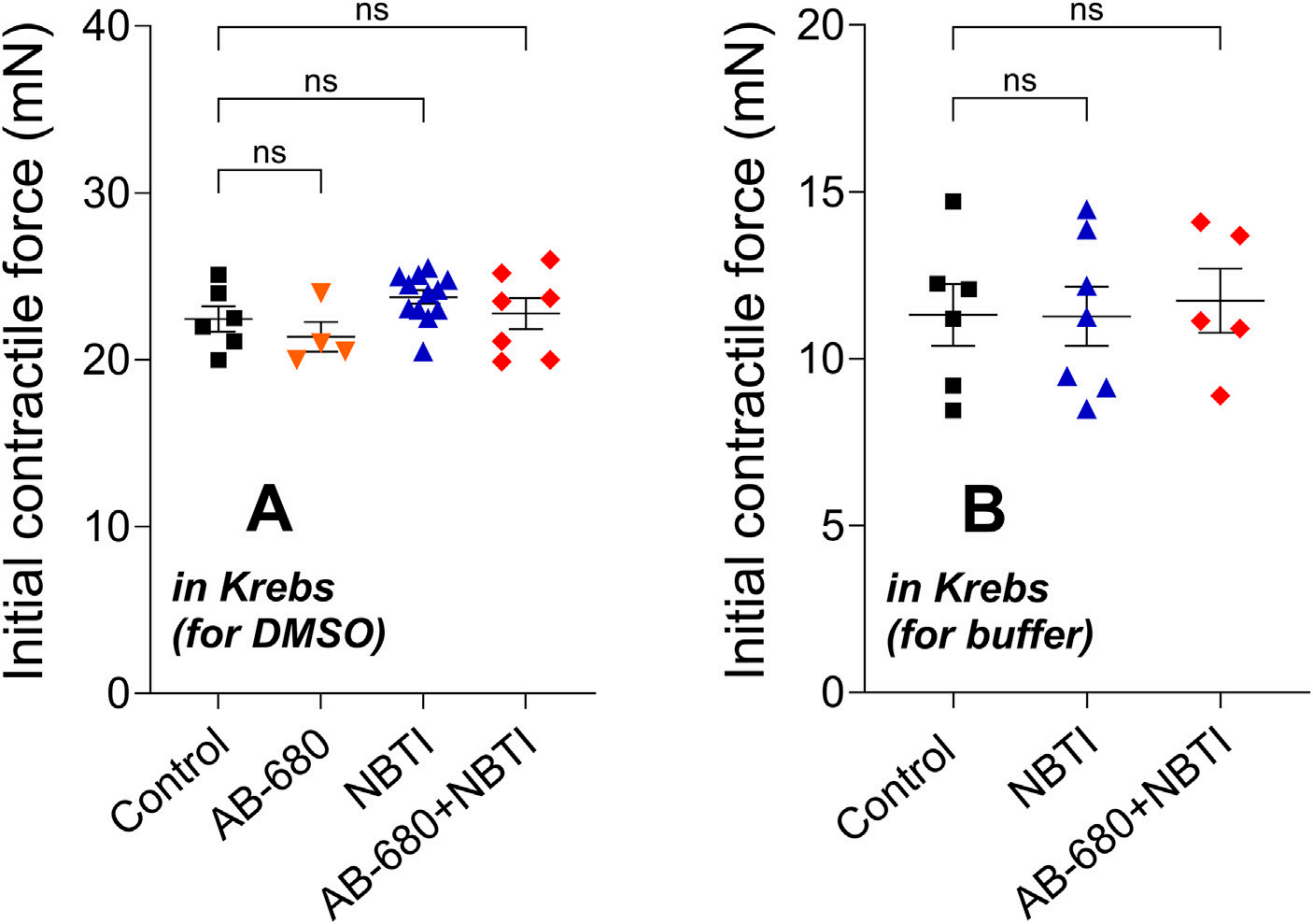
Initial contractile force of atria in the four [first series of experiments; panel **(A)**] or three [second series of experiments; panel **(B)**] groups (mean ± SEM) measured for the adenosine E/c curve generated in the absence of any inhibitors and their solvents. The color code used for the groups is uniform for all figures in this work. E/c, concentration–effect; Krebs, Krebs–Henseleit solution; DMSO and buffer, solvents administered subsequently (but not here); NBTI, S-(2-hydroxy-5-nitrobenzyl)-6-thioinosine; ns, statistically non-significant.

The initial contractile forces, which were measured before the construction of the adenosine E/c curve (in the absence of any solvents and inhibitors), did not differ significantly among the different groups in either the first (Figure 1A) or second series of experiments (Figure 1B).

### 3.2 Adenosine E/c curves

**FIGURE 2:**
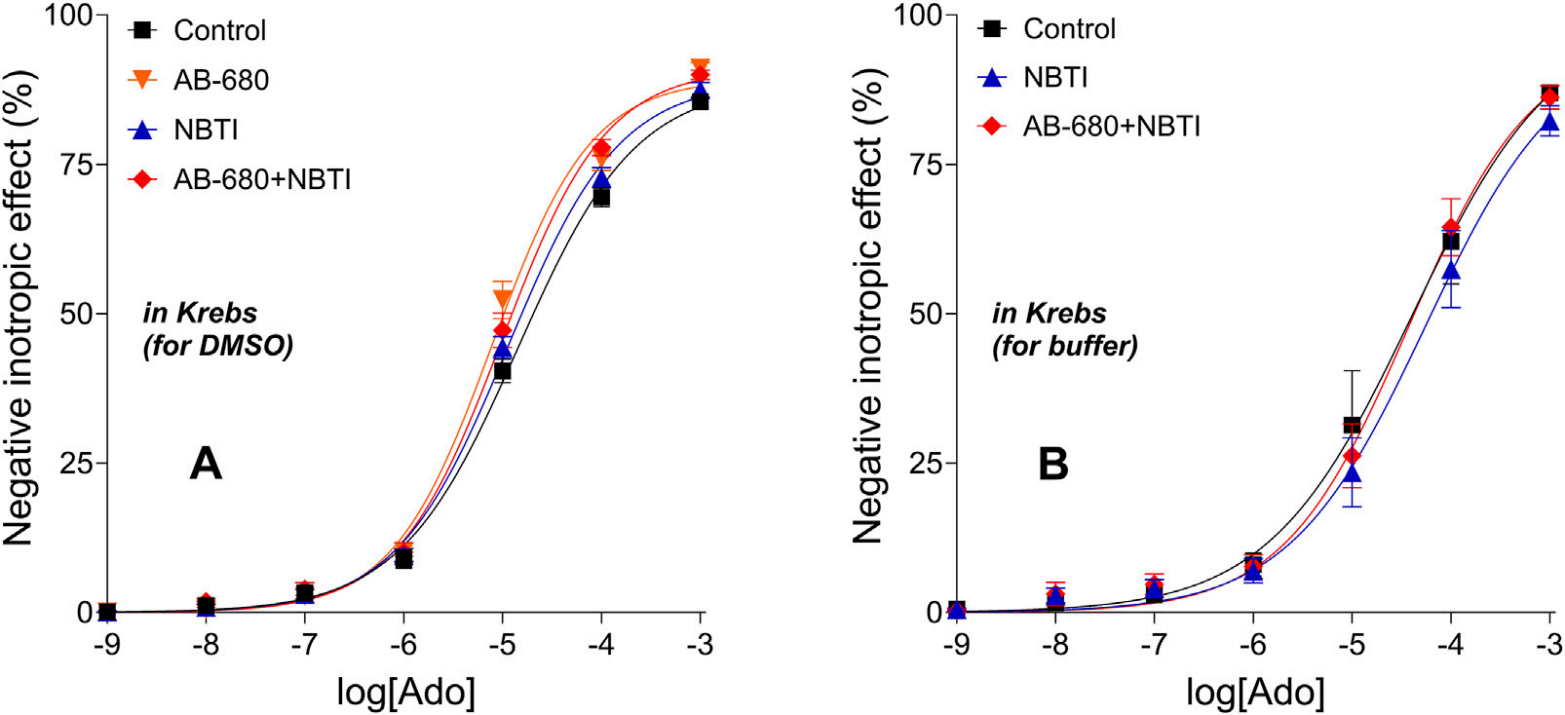
Direct negative inotropic effect of adenosine in the isolated guinea pig left atrium in the absence of any chemicals affecting the adenosinergic homeostasis (the names of the groups only refer to a subsequent exposure to the chemical(s) indicated). Panels **(A,B)** apply to the first and second series of experiments, respectively. The x-axis shows the common logarithm of the molar concentration of adenosine (used for the E/c curve), and the y-axis denotes the effect (as a percentage decrease in the initial contractile force). The symbols indicate the responses to adenosine averaged within the groups (±SEM). The continuous lines show the fitted Hill equation. The color code used for the groups is uniform for all figures in this work. E/c, concentration–effect; Ado, adenosine; Krebs, Krebs–Henseleit solution; DMSO and buffer, solvents administered subsequently (but not here); NBTI, S-(2-hydroxy-5-nitrobenzyl)-6-thioinosine.

The concentration-dependent, direct negative inotropic effects of adenosine showed no statistically significant difference among the groups within the first (Figure 2A) and second (Figure 2B) series of experiments. Thus, the atria used in the same series of experiments proved to form a homogenous population in terms of their response to adenosine, the endogenous agonist of the A_1_ adenosine receptor.

### 3.3 Effect of AB-680 and NBTI, alone and together, on the CPA E/c curves

#### 3.3.1 First series of experiments (carried out in the presence of DMSO, which is recommended for *in vitro* use as a solvent)

**FIGURE 3:**
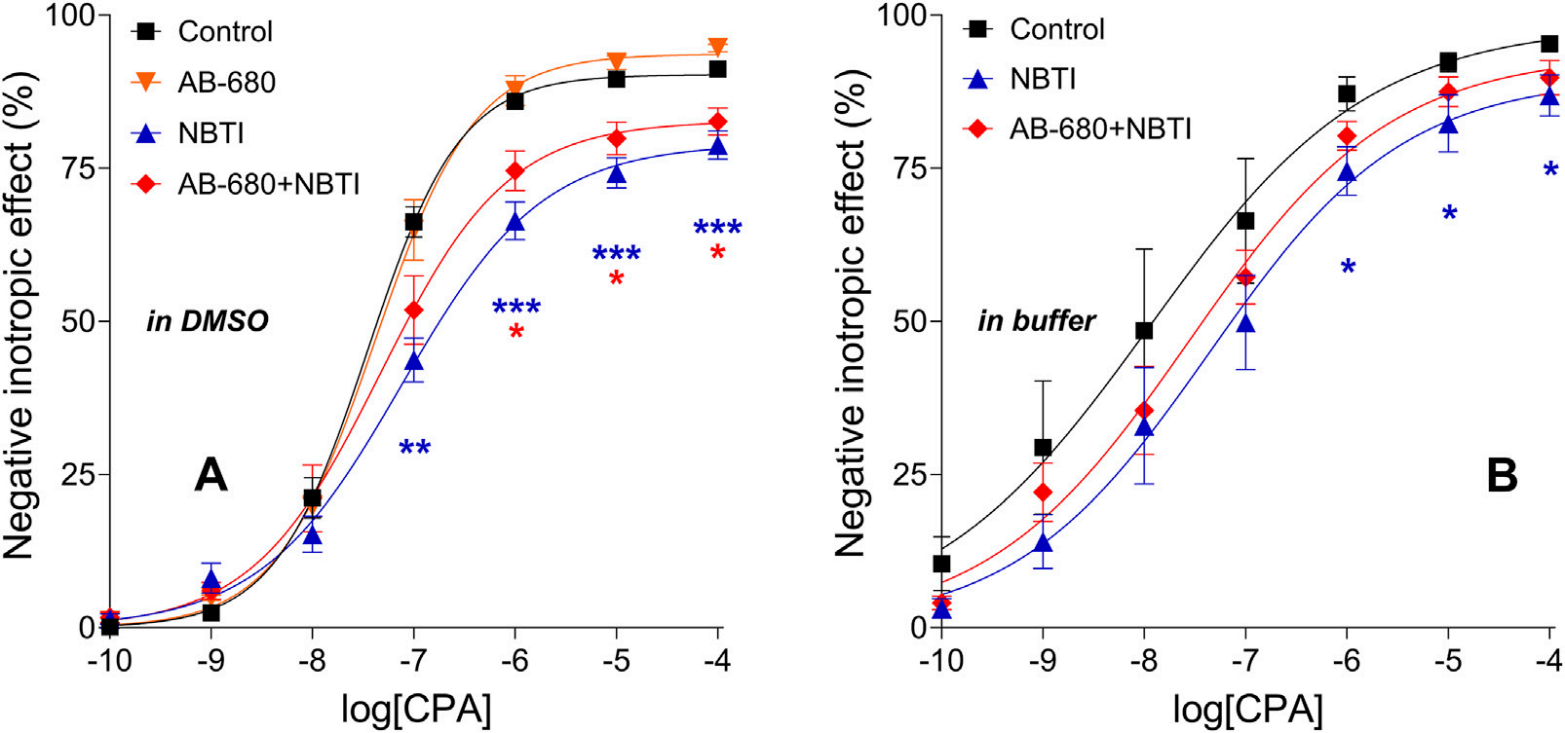
Direct negative inotropic effect of CPA in the isolated guinea pig left atrium in the absence and presence of 3 μM AB-680 and 10 μM NBTI (in all combinations). Panels **(A,B)** refer to the first (using DMSO as a solvent) and second (using a buffer as a solvent) series of experiments, respectively. The x-axis shows the common logarithm of the molar concentration of CPA (used for the E/c curve), while the y-axis denotes the effect (as a percentage decrease in the initial contractile force). The symbols indicate the responses to CPA averaged within the groups (±SEM). The continuous lines show the fitted Hill equation. The color code used for the groups is uniform for all figures in this work. E/c, concentration–effect; CPA, *N* ^*6*^-cyclopentyladenosine; DMSO, dimethyl-sulfoxide (recommended to dissolve AB-680 when used for *in vitro* experiments); buffer, a mixture of chemicals presented in the 2.1 subsection (recommended to dissolve AB-680 when used for *in vivo* experiments); NBTI, S-(2-hydroxy-5-nitrobenzyl)-6-thioinosine; blue asterisks, comparison between control and NBTI groups; red asterisks, comparison between control and AB-680 + NBTI groups; one asterisk, *p* < 0.05; two asterisks, *p* < 0.01; three asterisks, *p* < 0.001.

CPA also exerted concentration-dependent, direct negative inotropic effects in all groups. NBTI, as expected, significantly reduced the maximum of the CPA E/c curve. In contrast, AB-680, when administered alone, did not significantly affect the response to CPA. However, AB-680 co-administered with NBTI considerably reversed the suppressive action of NBTI on the CPA E/c curve (Figure 3A). In a direct comparison (AB-680 + NBTI vs. NBTI), this effect proved not to be statistically significant, but when using an indirect approach (comparing the levels of statistical significance for control vs. AB-680 + NBTI and control vs. NBTI), it appeared meaningful, although moderate (Figure 3A).

**FIGURE 4:**
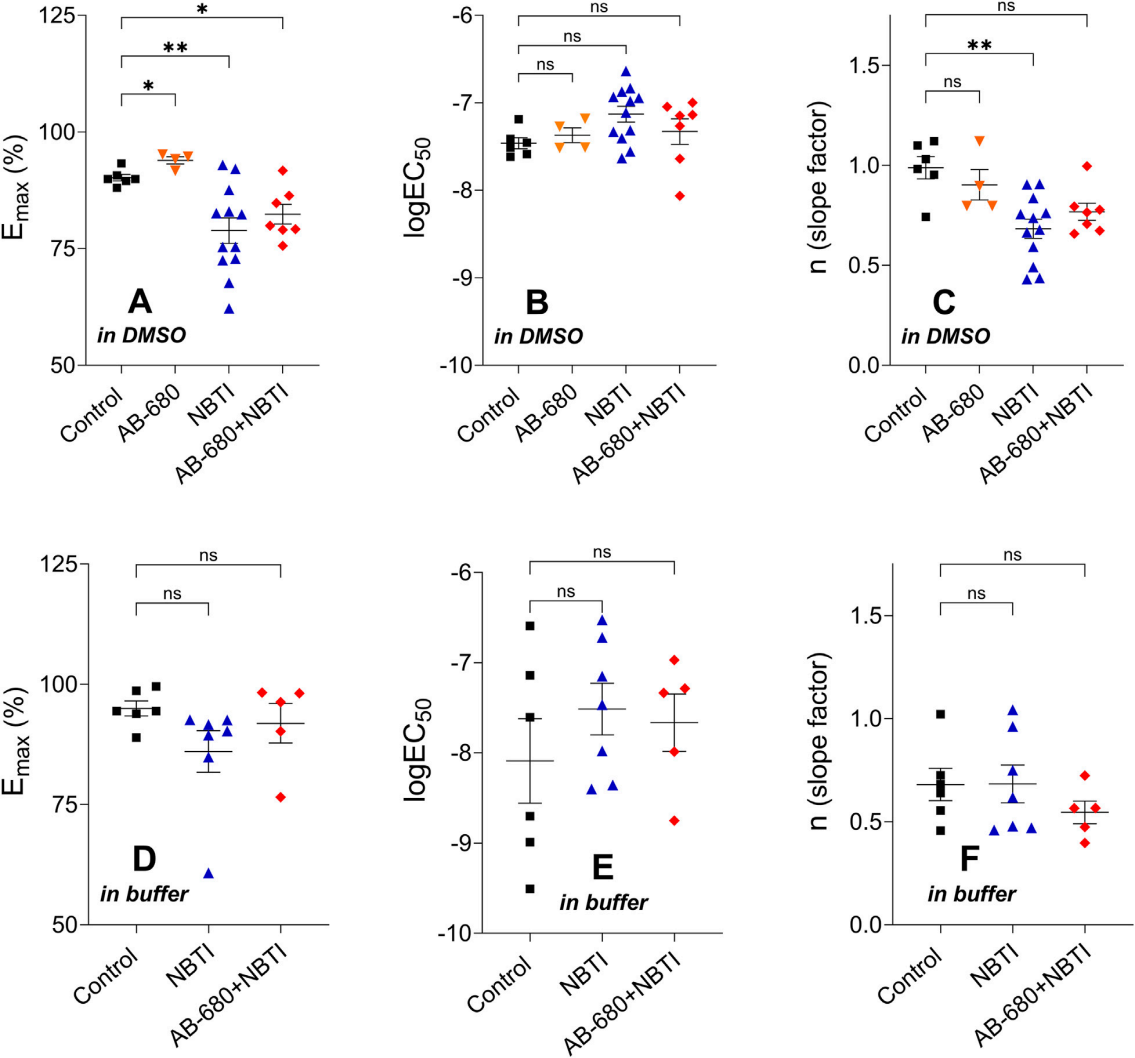
Hill parameters (E_max_, logEC_50_, and n) of the CPA E/c curves in the four [first series of experiments; panels **(A–C)**] or three [second series of experiments; panels **(D–F)**] groups (mean ± SEM). The color code used for the groups is uniform for all figures in this work. In terms of the corresponding Hill parameters, the treated groups were compared to the appropriate control group. E/c, concentration–effect; CPA, *N* ^*6*^-cyclopentyladenosine; DMSO, dimethyl-sulfoxide (recommended to dissolve AB-680 when used for *in vitro* experiments); buffer, a mixture of chemicals presented in the Subsection 2.1 (recommended to dissolve AB-680 when used for *in vivo* experiments); NBTI, S-(2-hydroxy-5-nitrobenzyl)-6-thioinosine; one asterisk, *p* < 0.05; two asterisks, *p* < 0.01; ns, statistically non-significant.

The E_max_ values determined from the CPA E/c curves of the treated groups of the first series of experiments significantly differed from the control E_max_. Although the maximal effect increased in the AB-680 group, it decreased in the NBTI and AB-680 + NBTI groups. Regarding the logEC_50_ values, the differences did not reach the level of statistical significance. Additionally, the Hill coefficient (n) in the NBTI group was significantly lower than the control value (Figures 4, A–C).

#### 3.3.2 Second series of experiments (performed in the presence of a buffer, which is recommended for *in vivo* use as a solvent)

When the buffer was used to dissolve NBTI and AB-680, the effect of NBTI on the CPA E/c curve, both alone and in combination with AB-680, showed the same pattern as when DMSO was applied (Figure 3B). However, in the presence of the buffer, the scatter of the data was larger, the CPA E/c curves were closer to each other, and even the shape of the CPA E/c curves was somewhat different compared to that of the case when DMSO was present as a solvent (*cf.* Figures 3A, B). Statistically, only the control and NBTI groups differed significantly from each other (Figure 3B), but it was to a lesser extent than in the presence of DMSO (Figure 3A).

The corresponding Hill parameters of the CPA E/c curves did not show any significant differences among the groups of the second series of experiments (Figures 4, D–F).

### 3.4 Influence of AB-680 on the surplus interstitial adenosine induced by NBTI

In the first series of experiments (using DMSO), c_x_, the CPA concentration equieffective with the surplus endogenous adenosine concentration produced by NBTI, was approximately 2.2-fold and 2.5-fold higher in the NBTI group than in the AB-680 + NBTI group when assessed using RRM implemented with individual fitting and two-model global fitting, respectively (Table 1). In turn, the c_x_ values determined with individual fitting were approximately 2-fold higher than the corresponding c_x_ values obtained with two-model global fitting (Table 1) (we made sure that when comparing c_x_ values, they only differed in one aspect).

In contrast, in the second set of experiments (using the buffer), c_x_ was approximately 6.7-fold and 6.3-fold higher in the NBTI group than in the AB-680 + NBTI group when obtained with individual fitting and two-model global fitting, respectively (Table 1). Nevertheless, the c_x_ values determined with individual fitting were approximately 2.5-fold higher than the corresponding c_x_ values obtained with two-model global fitting, which was similar to the outcome observed in the first series of experiments (Table 1). However, when comparing the corresponding c_x_ values between the two series of experiments, approximately 40–140-fold (!) higher c_x_ values were obtained when DMSO was used as a solvent (Table 1).

**FIGURE 5:**
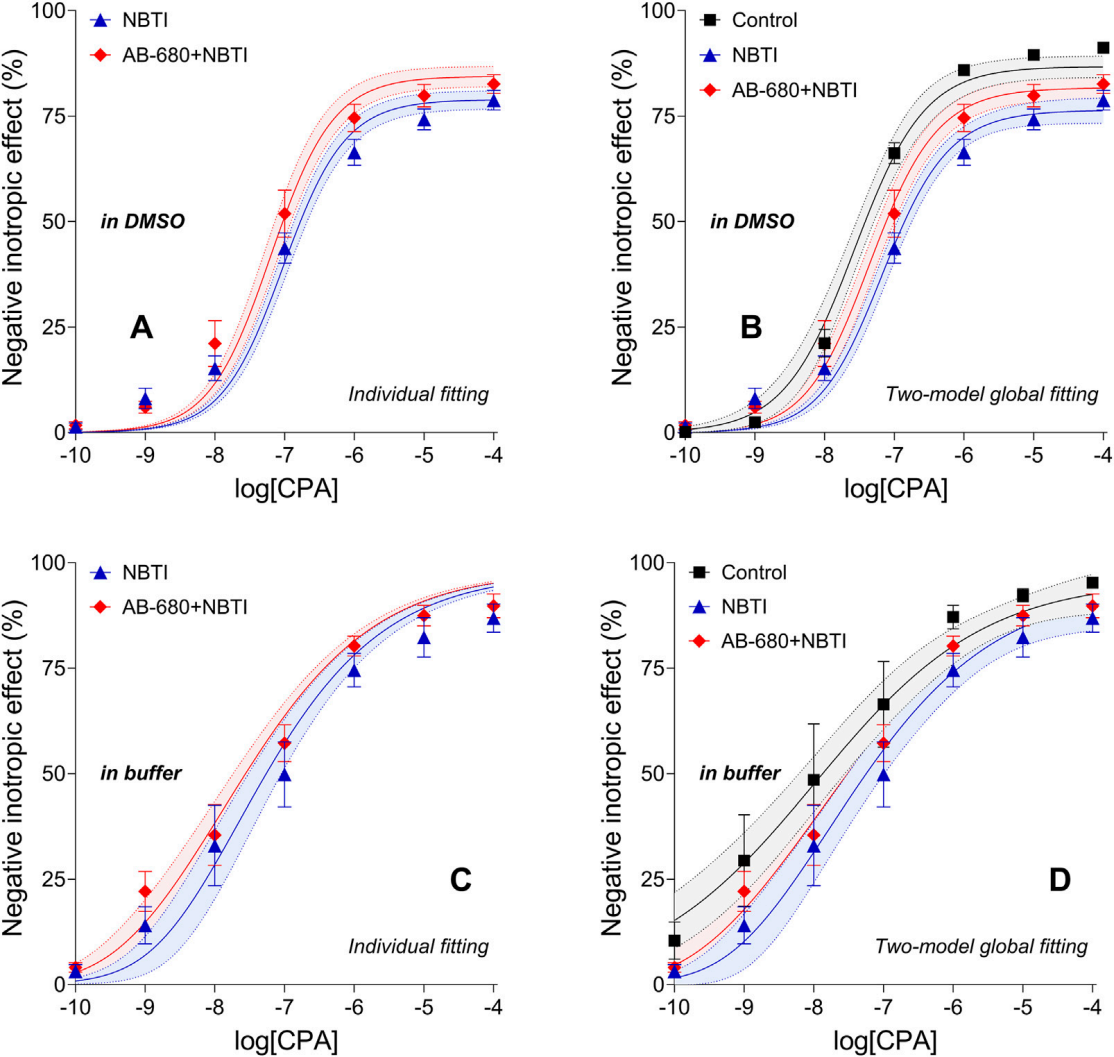
CPA E/c curves of the first [panels **(A,B)**] and second [panels **(C,D)**] series of experiments (except the AB-680 group) evaluated with RRM to obtain the CPA-equivalents of the surplus interstitial adenosine concentration caused by NBTI (i.e., c_x_ values). The x-axis shows the common logarithm of the molar concentration of CPA, and the y-axis denotes the effect. The symbols indicate the responses to CPA averaged within the groups (±SEM). The continuous lines denote the model of RRM fitted individually [panels **(A,C)**] or globally using two equations [panels **(B,D)**]. The dotted lines indicate the 95% confidence bands. The color code used for the groups is uniform for all figures in this work. E/c, concentration–effect; CPA, *N* ^*6*^-cyclopentyladenosine; DMSO, dimethyl-sulfoxide (recommended to dissolve AB-680 when used for *in vitro* experiments); buffer, a mixture of chemicals presented in the Subsection 2.1 (recommended to dissolve AB-680 when used for *in vivo* investigations); NBTI, S-(2-hydroxy-5-nitrobenzyl)-6-thioinosine.

Based on the 95% confidence bands indicating the precision of the curve fitting, the outcome of RRM, performed with both types of regression, appeared to be considerably more reliable when DMSO (and not the buffer) was used as a solvent. In the case of DMSO, the 95% confidence bands were clearly separated from each other, especially in the middle and terminal portions (individual fitting) or in the middle section (two-model global regression) of the CPA E/c curves (Figures 5A,B). Contrary to this, for the buffer, the 95% confidence bands largely overlapped (Figures 5C,D). Consequently, DMSO, a solvent widely used for *in vitro* investigations, interfered with the determination of c_x_ to a lesser extent than the buffer recommended as a solvent for *in vivo* studies (MCE, 2025).

In summary, in our *ex vivo* experimental setup, following the dissolution protocol of AB-680 recommended for *in vitro* use (MCE, 2025), AB-680 reduced c_x_, a measure of the interstitial adenosine accumulation caused by NBTI, by at least half. This outcome suggests that in response to AB-680, the concentration of the surplus interstitial adenosine accumulated by NBTI may also have been halved.

## 4 Discussion

In the present study, we found that AB-680, a highly potent CD73 inhibitor, did not affect the atrial direct negative inotropic response to CPA, a selective A_1_ adenosine receptor full agonist, when administered alone. In turn, when added together with NBTI, a nucleoside transport blocker, AB-680 was able to partially reverse the effect of NBTI on the response to CPA. Nevertheless, the inhibitory action of AB-680 on the effect of NBTI appeared to be less than that of PSB-12379, a less potent CD73 inhibitor, which was investigated earlier in the same experimental model (Viczjan et al., 2021). We also found that to dissolve AB-680 for our *ex vivo* investigation, the protocol recommended for *in vitro* use was preferable to that recommended for *in vivo* use (i.e., the simple DMSO should be preferred over the complex buffer). In addition, AB-680, co-administered with NBTI, reduced c_x_, the CPA-equivalent of the surplus interstitial adenosine concentration, by at least half (when DMSO was used as a solvent).

Adenosine exerts a variety of protective and regenerative effects in multicellular organisms (Fredholm et al., 2001; Fredholm et al., 2011; Burnstock et al., 2010; IJzerman et al., 2022). In the mammalian myocardium, all four adenosine receptor (sub)types are expressed and may influence cardiac function, including contractility (Chandrasekera et al., 2010). Nevertheless, most of the direct (proximate) effects of adenosine on the myocardium are mediated by A_1_ and (to a lesser extent) A_3_ adenosine receptors (Fredholm et al., 2001; Szentmiklósi et al., 2011; Headrick et al., 2013; Qian et al., 2025). The significance of the A_2_ subtypes has been highlighted in relation to atrial fibrillation (Maille et al., 2021).

The most striking acute cardiac actions of adenosine are the negative tropic effects, including negative inotropy (Headrick et al., 2013; Burnstock and Pelleg, 2015; Qian et al., 2025). Due to the extracellular location of the binding site of adenosine receptors, the interstitial level of adenosine plays a prominent role in the adenosinergic mechanisms in all organs, including the heart (Szentmiklósi et al., 2011; Headrick et al., 2013; Burnstock and Pelleg, 2015; Qian et al., 2025). Consequently, any agent influencing the interstitial adenosine levels should also be examined from this perspective.

The negative inotropic effect is particularly strong in the supraventricular myocardium (compared to the ventricular one), where, in most species, including the guinea pig and human, adenosine can significantly reduce the contractile force in a direct manner, thus without any previous positive inotropic stimulation (Belardinelli et al., 1995; Fredholm et al., 2001). The significance of atrial contractility stems from the fact that its decrease worsens the atrial booster pump function and, thereby, the ventricular filling (Rossi et al., 2000; Hoit, 2014). Moreover, the reduced atrial pumping capacity enhances the risk of atrial thrombus formation (Beigel et al., 2014; Yilmaz et al., 2020). Thus, it is important to consider atrial contractility (in addition to ventricular contractility) when investigating drugs or drug candidates that can (potentially or in a proven manner) influence the cardiac adenosinergic system.

In a previous study, we found that PSB-12379, a potent inhibitor of CD73, a key enzyme of the extracellular adenosine formation (Bhattarai et al., 2015; Schmies et al., 2020), significantly blunted the suppressing effect of NBTI on the response to CPA in an *ex vivo* guinea pig left atrium model (Viczjan et al., 2021). The transformation of the CPA E/c curve caused by NBTI (a decrease in the maximal effect and potency) was ascribed to the elevating effect of NBTI on the interstitial adenosine level (Gesztelyi et al., 2003; Karsai et al., 2006; 2007; Erdei et al., 2018; Viczjan et al., 2021). In the present work, we obtained a similar result using AB-680, an extremely potent CD73 inhibitor (Bowman et al., 2019), in the same experimental model (Figure 3). Nevertheless, there is an interesting difference between the previous and present results; i.e., the influence of PSB-12379 on the NBTI’s effect appears to be stronger than that of AB-680 (*cf.* Figure 6, left panel in Viczjan et al., 2021 with Figure 3A in this work). This difference suggests that, at least in our model, AB-680 affects the myocardial adenosinergic mechanisms to a lesser extent than PSB-12379, another CD73 inhibitor, does. Greater potency (*cf.* the picomolar affinity of AB-680 (Bowman et al., 2019) and the nanomolar affinity of PSB-12379 (Bhattarai et al., 2015) for CD73) does not necessarily mean greater efficacy (maximal effect) (Brunton and Knollmann, 2023). Regarding the potential cardiac side-effects of AB-680, this result is more favorable than undesirable.

It should also be addressed that AB-680, similarly to PSB-12379 (Viczjan et al., 2021), did not significantly affect the CPA E/c curve when administered alone (Figure 3A). This may be because under our experimental circumstances, the naïve interstitial adenosine level did not appear to be capable of activating the atrial A_1_ adenosine receptors (Pak et al., 2015). Hence, it is not surprising that a decrease in an already ineffective adenosine level could not influence the response to CPA (Figure 3A).

AB-680 was reported to have a slow onset regarding the inhibition of CD73. Increasing the pre-incubation time of human CD73 with AB-680 resulted in decreased IC_50_ values, with the plateau reached at approximately 30 min (Bowman et al., 2019). The 30-min incubation period used in the present investigation addressed this previous observation.

Regarding c_x_, the CPA concentration equieffective with an increase in the atrial interstitial adenosine concentration caused by NBTI, AB-680 reduced its value by at least half when added together with NBTI (and DMSO was used as a solvent) (Table 1). The c_x_ values provided by RRM are unique in that they characterize the increase in the adenosine concentration in the microenvironment of the binding sites of myocardial A_1_ adenosine receptors, which is a tissue compartment that is otherwise difficult to assess in this regard (Karsai et al., 2006; 2007; Erdei et al., 2018). In light of our previous results, the current c_x_ values (determined for NBTI alone, using DMSO), 52.65 nM and 27.39 nM (obtained with individual fitting and two-model global fitting, respectively; Table 1), can be considered average (Erdei et al., 2018).

In addition, DMSO, recommended as a solvent for AB-680 under *in vitro* conditions (MCE, 2025), interfered with our *ex vivo* measurements to a lesser extent than the buffer recommended to dissolve AB-680 in *in vivo* studies (MCE, 2025) (*cf.* Figure 3A with Figure 3B; Table 1). Therefore, for future *ex vivo* studies, we suggest using DMSO for dissolving AB-680 (or chemicals with a similar solubility profile).

## Data Availability

The raw data supporting the conclusions of this article will be made available by the authors, without undue reservation.
